# Hiring qualified Forensic Investigative Genetic Genealogy (FIGG) practitioners: Recommendations for forensic science and law enforcement agencies

**DOI:** 10.1016/j.fsisyn.2026.100688

**Published:** 2026-05-19

**Authors:** Wendy McLean, Cathryn Fahey, Stacey Baker, Allison Ryall, Rhonda Kevorkian, Daren Boyd, Andrea McCarthy, Sideny Collins, Dana Philipps, Jeffrey Baney

**Affiliations:** Center for Human Identification at UNT Health Fort Worth, United States; Idaho State Police Forensic Services, United States; Palm Beach County Sheriff's Office Crime Laboratory, United States; BODE Technology, United States; Compass IGG & Advocacy, United States; Idaho State Police Forensic Services, United States; Resolve Forensics, United States; Massachusetts State Police Crime Laboratory, United States; Union County Prosecutors Office Forensic Laboratory, United States; Pennsylvania State Police Bureau of Criminal Investigation, United States

## 1 Introduction

The National Technology Validation and Implementation Collaborative (NTVIC) was established in 2022 with a vision to collaborate nationally on forensic science validation, method development, and implementation in the public sector. The subcommittee for Public Entity Investigative Genetic Genealogist was formed in 2025 with the mission to promote robust, reliable, and ethical practice of Investigative Genetic Genealogy in the public sector in collaboration with subject matter experts across various fields including but not limited to forensic science, law, traditional genealogy, law enforcement, and bioethics.

While this document was prepared by members of the Public Entity Investigative Genetic Genealogist Subcommittee, inclusion of an individual's name does not imply full agreement with every recommendation or statement. The content represents a consensus of perspectives and contributions from the group.

## 2 Recommendations

As the field of Forensic Investigative Genetic Genealogy (FIGG) continues to expand, there is increasing value in hiring an in-house FIGG practitioner in the public sector. Public entities such as law enforcement agencies, forensic laboratories, and prosecutors’ offices, are seeking in-house FIGG Practitioners to enhance confidentiality and data security, ensuring sensitive genetic and case information is carefully managed. Qualified FIGG practitioners bring a wealth of practical experience and capability. Employing FIGG practitioners internally also improves speed and efficiency by allowing the employing agency to prioritize case work according to agency goals, track case progress, and anticipate additional investigative work. Increased speed and efficiency will result in faster case turnaround times which will, in turn, lead to greater cost-effectiveness over time. In-house FIGG practitioners facilitate more integrated workflows by working closely with lab scientists, law enforcement and medicolegal authority.

The growing role of FIGG in unresolved cases and violent crimes highlights the necessity of having skilled in-house professionals capable of genetic genealogy. However, hiring a FIGG practitioner requires identifying a candidate with a combination of working knowledge of genetic inheritance, experience in the field of genealogy, critical thinking skills, and ethical integrity. The following recommended qualifications are categorized into responsibilities, education, experience, knowledge and skills to consider, where applicable, when choosing a professional in this evolving field.

## 3 Responsibilities

### 3.1 Required

• Perform independent research to identify an unknown subject or unidentified human remains using information found in accessible genetic genealogy databases.
• Use publicly available records (birth, marriage and death records; newspaper archives; census and church records) along with law enforcement data to develop family trees of genetic relatives of the unidentified person.
• Conduct public source descendancy research to identify family members at the appropriate genetic distances.
• Identify Most Recent Common Ancestors (MRCA) using methods such as clustering, triangulation, and chromosome mapping.
• Utilize verbal and written communication skills to communicate case status, findings, and recommendations for further research and findings to appropriate parties.
• Maintain strong working relationship with law enforcement built on professionalism.
• Maintain a high level of analytical ability through continuing education.
• Comply with policies procedures, rules, and regulations.
• Create standard operating procedures for genetic genealogical research and related activities, improving them over time, adhering to them and ensuring others are following the same approach.
• Create coherent, concise and thorough documentation of cases and activities.

### 3.2 Additional responsibilities as assigned

• Rank cases based on solvability factors (highest match, presence of clusters, biogeographical ancestry, availability of records)
• Provide education/training to agencies and in-house personnel
• Testify in court or administrative hearings
• Perform technical review of FIGG reports
• Offer recommendations on training, seminars, resource material, etc. to enhance the agency's capabilities
• Liaise with other FIGG practitioners
• Participate in working groups
• Attend ongoing training to stay abreast of new skills, technologies, techniques and requirements
  If the position includes duties traditionally assigned to a Criminalist or DNA analyst, additional responsibilities may include
• Review cases for FIGG eligibility
• Enter/update cases in applicable databases (NamUs, NCIC, etc.)
• Collaborate with evidence technicians and forensic scientists, regarding evidence, sample quality, and SNP technology options
• Coordinate and facilitate the upload of SNP profiles to eligible genetic genealogy databases.

To become a FIGG practitioner, individuals typically pursue a multidisciplinary path that blends genetics, genealogy, and criminal justice. While education may provide foundational knowledge, specialized training in genetic genealogy, through certificate programs or credentials from different organizations, develops research skills essential for tracing family histories. Further specialization in FIGG can be gained through targeted certificate programs.

### 3.3 Education

Preferred qualifications^1^.

• Bachelor's degree in related field or equivalent FIGG experience
• Satisfactory completion of a certificate from an educational organization with coursework including but not limited to:
• Fundamentals of forensic biology (introduction, biological evidence, types of DNA profiles, CODIS, SNP technologies)
• Genetic genealogy principles and methods (SNP technologies, privacy and ethical issues, match interpretation, inheritance patterns, genetic genealogy databases, tools/software for interpreting match data, building family trees)
• Genealogy principles and methods (documentation and research, records, genealogical proof standard)
• Traditional or genetic genealogy coursework certificate

## 4 Credentialing

When posting a job, employers should consider evaluating individuals credentialed by a recognized board as part of their candidate screening process. Credentialing serves as official recognition that a person has met established professional standards, providing a reliable way to identify qualified individuals and ensure they possess the necessary knowledge and skills for the role. FIGG practitioners pursue credentials for several reasons including gaining professional recognition and advancing their careers, which can represent a personal achievement that reflects dedication and expertise. Additionally, credentialing encourages ongoing education, helping professionals stay current in their fields and maintain high standards of excellence. Several recognized credentialing boards are available in the fields of genealogy and FIGG.

An ideal candidate for a position as a FIGG practitioner possesses a strong foundation in traditional genealogy along with advanced expertise in genetic genealogy. Many professionals in this field have developed their genetic genealogy skills through practical application with cases involving unknown or misattributed parentage—scenarios that closely resemble missing persons and forensic casework, rather than the more conventional use of genetic genealogy to confirm documented family trees. The most qualified candidates will have hands-on experience constructing and analyzing complex family trees, interpreting DNA results, documenting their work and findings and operating within the ethical and legal frameworks required in the field and public sector.

Below is a breakdown of the essential knowledge and skills to look for in a qualified candidate.

## 5 Knowledge areas

• Understands genetic inheritance patterns and average amounts of shared DNA between two people for close and distant relationships
• Understands segment triangulation and the tools used to identify genetic networks
• Familiar with using the amount of shared DNA to develop a hypothesis about where the genetic relatives might belong in the tree to guide tree building
• Knowledge of historical records, and where to find them in public genealogy databases, online archives, and physical locations (libraries and other repositories).
• Awareness of the limitations of public genealogy databases and genetic genealogy databases that allow use by law enforcement and those working on behalf of law enforcement
• Knowledge of which genealogical databases are approved for law enforcement use, including opt-in/opt-out policies for each
• Ability to assess whether third-party DNA testing is necessary and identify the most appropriate individuals for testing
• Strong understanding of ethical considerations in forensic investigative genetic genealogy (FIGG), including privacy, consent, and potential misuse of information
• Familiarity with the U.S. Department of Justice (DOJ) Interim Policy on the use of FIGG, National Technology Validation and Implementation Collaborative (NTVIC) publications and other relevant policies and publications
• Familiarity with the National Missing and Unidentified Persons System (NamUs)
• Basic understanding of short tandem repeats (STRs) and Combined DNA Index System (CODIS) as it relates to FIGG
• Understanding of autosomal DNA, Y-DNA, mtDNA, X-DNA
• Understanding of forensic sample attributes (degradation, mixtures, etc.)

## 6 Skills

• Clear and logical presentation of findings through oral reports, written documentation, charts, and diagrams
• Proficiency in word processing and spreadsheet software and applications
• Ability to concentrate for extended periods of time
• Effective team collaboration and meaningful contribution to group efforts
• Strict adherence to confidentiality and data security protocols
• Hands-on experience with classic GEDmatch and FamilyTreeDNA (FTDNA), including DNA file management and user guidance
• Experience using GEDmatch PRO
• Familiarity with genetic genealogy database tools used to interpret genetic relative data
• Self-motivated, independent, and organized work style
• Strong attention to detail and the ability to organize and manage large volumes of data
• Multitasking and project management capabilities
• Willingness to undergo interview-based testing or post-employment skills assessments (e.g., competency tests)
• OSINT (Open-Source Intelligence) skills

When evaluating candidates for hire, agencies may wish to consider several additional criteria. The position can be structured as either a contract or payroll role, depending on organizational needs. Applicants should be informed that signing a confidentiality agreement may be required. To assess skill level, agencies might review the candidate's years of experience and the number or type of cases they have handled. When possible, the organization may consider evaluating a candidate's skills by administering a timed practical exercise with a known result and/or reviewing a candidate's former work. A background check is typically part of the process. These considerations can help ensure a thorough and effective hiring strategy.

## CRediT authorship contribution statement

**Wendy McLean:** Conceptualization, Data curation, Project administration, Writing – original draft, Writing – review & editing. **Cathryn Fahey:** Conceptualization, Formal analysis, Writing – review & editing. **Stacey Baker:** Conceptualization, Data curation, Formal analysis, Methodology, Writing – original draft, Writing – review & editing. **Allison Ryall:** Data curation, Formal analysis, Resources, Writing – review & editing. **Rhonda Kevorkian:** Data curation, Formal analysis, Resources, Writing – review & editing. **Daren Boyd:** Formal analysis, Resources, Writing – review & editing. **Andrea McCarthy:** Data curation, Formal analysis, Resources, Writing – review & editing. **Sideny Collins:** Data curation, Formal analysis, Writing – original draft, Writing – review & editing. **Dana Philipps:** Resources, Writing – review & editing. **Jeffrey Baney:** Formal analysis, Resources, Writing – review & editing.

## Declaration of competing interest

The authors declare that they have no known competing financial interests or personal relationships that could have appeared to influence the work reported in this paper.

## Glossary

**Accessible Genetic Genealogy Databases**: a public or private genetic genealogy database where users upload their SNP profile and are given the explicit privacy option (opt-in/out) to have that data compared against the relevant forensic SNP profiles for the purposes of FIGG
**Autosomal DNA (atDNA)**: DNA found in the nucleus of a human cell and comprises the 22 pairs of non-sex chromosomes
**Biogeographical ancestry**: In direct-to-consumer testing an estimate of a person's ancestral origin compared to geographic population groups
**Chromosome mapping**: A visual representation of DNA segments shared between genetic relatives used in genetic genealogy to determine common ancestors between individuals who share the same DNA segments
**Clustering**: A method of sorting genetic relatives based on common ancestors and/or shared DNA segments
**Combined DNA Index System (CODIS)**: the FBI-managed DNA database system that allows federal, state and local law enforcement laboratories to store and compare DNA profiles from crime scenes, convicted offenders, and missing persons in order to identify potential suspects and generate investigative leads
**DNA**: deoxyribonucleic acid
**Genetic Relative:**: In genetic genealogy, a person who has taken a Direct-to-Consumer DNA test and appears in a list of other testers that share DNA with the primary test taker
**Descendancy research**: In genealogy, research into a person or couples' descendants. The research typically begins in the past and moves forward toward the present day
**Forensic Investigative Genetic Genealogy or FIGG**: a procedure that combines genetic testing with traditional genealogical research to generate leads in investigations of unsolved violent crimes and unidentified human remains
**Genetic inheritance**: The inheritance of genetic material that occurs during cell division. The four kinds of DNA used in genetic genealogy each have different genetic inheritance patterns
**Genetic networks**: Groups of genetic relatives that share DNA with everyone in the group.
**Misattributed parentage**: A situation where an individual's presumed parent is not their biological parent usually discovered during genetic testing
**Mitochondrial (mtDNA)**: A small circular DNA molecule located in the mitochondria that contains approximately 16,500 nucleotides. All children inherit their mtDNA from their mothers
**Most Recent Common Ancestors (MRCA)**: The closest ancestor who is shared by two or more individuals
**Opt-in/opt-out Database Privacy settings**: Privacy settings in a genetic genealogy database that allow the user to opt-in or opt-out of law enforcement matching
**Single nucleotide polymorphisms or SNP**: DNA sequence variations that occur when a single nucleotide (A, T, G or C) in a genomic sequence varies. SNPs include variations that may be used to distinguish people for purposes of biological relationship testing or direct comparison purposes
**SNP profile:**: A record of DNA sequence variants across an individual's genome for upload to a publicly accessible genetic genealogy database
**Technical review**: Evaluation, by a qualified individual, of all supporting records from the examination and the report, if prepared, to evaluate observations and assess whether there is an appropriate and sufficient basis for any opinions
**Third-party DNA testing**: DNA analysis conducted on a collected DNA sample from an individual with their consent who is not a suspect in an investigation, to further genealogical research to identify the putative perpetrator
**Triangulation**: A method of identifying common ancestors using a group of three or more individuals that share DNA with the primary test taker and each other
**Unidentified human remains or UHR**: Remains from a person whose identity has not been established
**Unknown parentage**: A situation where one or both parents of an individual is unknown. For example, a person who was adopted, doner conceived, a foundling, etc
**X-DNA**: DNA inherited from the X-chromosome. Females inherit X-DNA from both parents, males inherit X-DNA from their mother only. This unique inheritance pattern can be useful in genetic genealogy
**Y-DNA**: DNA inherited from the Y-chromosome by males from their father

## Footnotes

1 Traditional genealogy and genetic genealogy education is often self-directed. Genealogists acquire education through genealogical societies that offer conferences, seminars and institute courses. Institute courses are one-week courses taught by a team of professional genealogists. Degrees in genealogy are limited.

